# Extended Inflammation Parameters (EIP) as Markers of Immune System Cell Activation in Psoriasis

**DOI:** 10.1155/2021/9216528

**Published:** 2021-06-14

**Authors:** Anna Kowalska-Kępczyńska, Mateusz Mleczko, Weronika Domerecka, Marcin Mazurek, Dorota Krasowska, Teresa Małecka-Massalska, Helena Donica

**Affiliations:** ^1^Department of Biochemical Diagnostics, Chair of Laboratory Diagnostics, Medical University of Lublin, Lublin, Poland; ^2^Chair and Department of Dermatology, Venerology and Paediatric Dermatology, Medical University of Lublin, Lublin, Poland; ^3^Chair and Department of Human Physiology, Medical University of Lublin, Lublin, Poland

## Abstract

Psoriasis is an inflammatory, autoimmune disease that affects approximately 2% of the population. The inflammation in psoriasis can be systemic, so despite a predominantly cutaneous manifestation, it also affects the internal organs. The diagnosis and monitoring of the disease are based on the clinical picture. To assess the disorders of other organs, additional tests need to be performed. Recently, the examination of blood morphology has been enriched with modern haematological parameters, i.e., Extended Inflammation Parameters (EIP), which include RE-LYMPH (activated lymphocytes), AS-LYMPH (antibody-producing B lymphocytes), and NEUT-RI and NEUT-GI (activated neutrophils). In the study, higher values of new haematological parameters were observed in individuals with psoriasis than in healthy controls. A higher EIP value was noted in the group of individuals with plaque psoriasis than in the group of individuals with psoriatic arthritis. Implementation of these parameters into routine laboratory analysis will likely make it possible to estimate the severity of the inflammation and improve its assessment.

## 1. Introduction

Psoriasis is the most common genetically determined skin condition, which affects about 2% of the population [1]. The inflammatory process, especially in mild forms of the disease, affects only the skin, manifesting itself in well-delimited plaques covered with silvery scales. Depending on the severity, the disease process may affect a limited area of the skin, but sometimes, the whole skin is affected [2]. In moderate and severe forms, the process is not limited to the skin and can affect other tissues and organs [3].

The classic example of involvement by inflammation of tissues other than skin is joints. Psoriatic arthritis is inflammation of the joints or synovium that affects up to 25% of psoriasis patients [4].

In recent years, in addition to psoriatic arthritis, metabolic syndrome, and thus cardiovascular diseases, has been included in the diseases accompanying psoriasis [5]. The exact underlying mechanism of metabolic syndrome in psoriasis has not been explained to date. The studies carried out so far have demonstrated inflammatory changes within the blood vessels of individuals with psoriasis and confirmed the correlation between the severity of involvement of blood vessels and psoriasis [3].

The changes observed in psoriasis result from an increased humoral and cellular response of the body to the triggering stimulus. This stimulus can be stress, infection, medication, improper diet, or stimulants. As a result of the noxious stimulus, dendritic cells (DCs) are stimulated, which start to present antigens to the auxiliary T lymphocytes (Th). Stimulated T lymphocytes—mainly Th-1, Th-17, Th-22, or Th-8—play a significant role in the mechanism of psoriasis development. As a result of activation, these cells release IL-17, IL-22, INF*γ*, and TNF*α*, which, on the one hand, directly stimulate the proliferation of keratinocytes and, on the other hand, induce the secretion of dendritic cells IL-12, IL-20, and IL-23. These cytokines force indirectly excessive cell division in the basal layer of the epidermis and accelerate abnormal maturation of keratinocytes. Stimulated epidermal cells release many proinflammatory substances (IL-8, CXCL-1, S100A7, S100A8, S100A9, S100A12, PDGF, VEGF, heat-shock proteins, etc.). As a result of these processes, the order of the skin layers is lost, and numerous cells of the immune system (lymphocytes, monocytes, and granulocytes) accumulate in the skin, which then move to the epidermis (the so-called Munro's microabscesses) [6].

The assessment of psoriasis is based almost exclusively on the clinical picture. Only in doubtful situations, especially when there is a lack of therapeutic efficacy, a histopathological examination of a section taken from a skin lesion is helpful. The assessment of the severity of the skin lesions is carried out using PASI (Psoriasis Area and Severity Index) and BSA (Body Surface Area) questionnaires, and the quality of life is assessed by means of the Dermatology Life Quality Index (DLQI scale), which is an indicator of the quality of life depending on the skin condition [7]. In case of changes in the joints, it is necessary to perform imaging examinations of the occupied area. More and more attention is also being paid to the analysis of individual components of the metabolic syndrome (arterial pressure, fasting glucose level, insulin, total cholesterol, HDL and LDL cholesterol, triglycerides, and WHR measurement) [5].

Considering the systemic nature of psoriasis, it seems to be of significant importance to look for a less tissue-specific marker of inflammation severity. The main objective of the study was to search for new markers of activation of cells responsible for the immune response. For this purpose, in a group of patients with psoriasis, the Extended Inflammation Parameters (EIP) were indicated including descriptors such as RE-LYMPH (activated lymphocytes), AS-LYMPH (antibody-producing activated lymphocytes), and NEUT-RI and NEUT-GI (activated neutrophils).

In recent years, the study of EIP has been almost exclusively limited to the assessment of these parameters in relation to bacterial and viral diseases, including publications on dengue fever, murine typhus, salmonellosis, leptospirosis, and malaria infections [8–12], and in recent months also COVID-19 infection [13, 14]. These studies show that a higher increase of RE-LYMPH and AS-LYMPH parameters is a characteristic for viral infections than for bacterial diseases. At the same time, it was shown that, in the case of bacterial aetiology, a greater increase in the NEUT-GI and NEUT-RI parameters is observed [15]. Equally interesting were the results of the study of parameters from the EIP group in patients with sepsis [16–19]. The increase in these parameters in this group of patients may indicate the activation of neutrophils and lymphocytes and may be useful in the detection of patients with sepsis in combination with the current biomarkers of sepsis. Despite the promising results of the lymphocyte and neutrophil activation studies presented above, the EIP in autoimmune diseases such as plaque psoriasis and psoriatic arthritis have not been studied so far. For this reason, it is worth emphasizing the innovation and the possibility of applying in diagnostic practice the data presented in this article.

## 2. Materials and Methods

### 2.1. Characteristics of Patients

31 patients from the Chair and Department of Dermatology, Venerology and Paediatric Dermatology of Independent Public Clinical Hospital No. 1 in Lublin, were included in the study. The study group consisted of 9 women and 22 men over the age of 18, who met the criteria for inclusion in the study. These were patients with diagnosed plaque psoriasis and psoriatic arthritis (see Table 1, column 2). The control group consisted of healthy individuals, represented by 30 volunteers over 18 years old. Cigarette smoking was declared by 8 patients (3 in the psoriasis group and 5 in the control group). Concomitant chronic diseases occurred in 28 patients (18 in the psoriasis group and 10 in the control group). None of the study participants suffered from cancer. Immunosuppressive therapy for psoriasis was currently used by 22 patients (12 patients treated with methotrexate, 5 patients with cyclosporine, and 5 patients with immunomodulatory drugs). The remaining patients were treated only topically. The absence of diagnosed inflammation and correct results of routine laboratory tests (see Table 1, column 3) were the absolute criteria for inclusion in the study in this group. Venous blood samples of 10 mL each were collected from all subjects using disposable equipment. Then, within 2 hours of collection, blood concentrations of selected haematological and biochemical parameters were determined. The study was approved by the Bioethics Committee at the Medical University of Lublin, obtaining permission to its implementation by resolution no. KE-0254/232/2019.

### 2.2. Apparatus and Methodology

Haematological determinations were performed with the use of Sysmex XN-1500 apparatus, while biochemical determinations were performed with the use of Roche Cobas Integra 800 apparatus and Multiskan FC microplate spectrophotometer with automatic microplate washer.

Serum and whole blood obtained from the ulnar vein were the material for the study. Blood was collected in the morning from fasting patients. First, blood samples (approx. 7.6 mL each) were collected in vacutainer serum clot activator tubes sized 8–16 × 50–100 mm to perform biochemical determinations. Then, the material was collected in 2.7 mL vacutainer tubes containing K3EDTA anticoagulant to determine the concentrations of selected haematological parameters. The test tubes were left to clot for approximately 20–30 minutes and subsequently centrifuged at 2500 rpm for 10 minutes. The whole blood sample for haematological tests, collected to K3EDTA, was analysed within 2 hours after sampling.

The examined haematological parameters include WBC (white blood cells), RBC (red blood cells), NEUT (neutrophils), and LYMPH (lymphocytes). New laboratory indicators of inflammation, the so-called EIP, were also analysed including parameters such as RE-LYMPH (reactive lymphocytes), AS-LYMPH (antibody-secreting reactive lymphocytes), NEUT-RI (neutrophil reactive intensity), and NEUT-GI (neutrophil granularity intensity). Besides, the erythrocyte sedimentation rate (ESR) was also measured. The assessed biochemical markers in the examined patients included the CRP level (C-reactive protein), IFN*γ* concentration (interferon gamma), and ANGII concentration (angiotensin II).

### 2.3. Statistical Methods

The statistical analysis of the test results was carried out using Statistica 13, StatSoft's statistical software. The statistical significance of the differences in the parameters was assessed by means of Student's *t*-test. The conclusion error was assumed to be 5%, and the associated statistical significance level is *p* < 0.05, which indicates the occurrence of statistically significant differences or relationships.

## 3. Results and Discussion

### 3.1. Results

Test results were developed using the basic elements of descriptive statistics.

Higher values of new haematological parameters (NEUT-RI, NEUT-GI, and RE-LYMPH) were observed in individuals with psoriasis than in healthy controls. Higher values of the aforementioned parameters were noted in the group of patients with plaque psoriasis than in the group of people with psoriatic arthritis (see Table 2, lines 1–3). No changes in the value of the AS-LYMPH parameter were observed (see Table 2, line 4).

**Figure 1:**
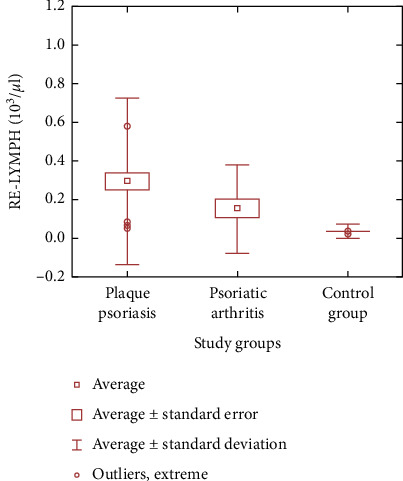
Differences in mean values of RE-LYMPH (reactive lymphocytes) in the study groups and the control group.

**Figure 2:**
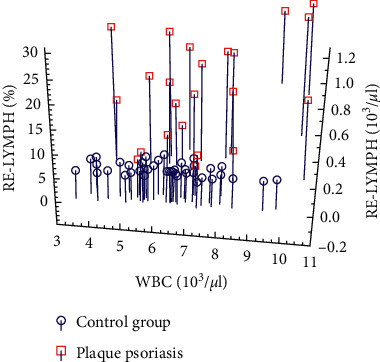
Relationship between RE-LYMPH%/WBC/RE-LYMPH# in the study group and the control group (RE-LYMPH#: absolute number of reactive lymphocytes; RE-LYMPH%: percentage of reactive lymphocytes; WBC: white blood cells).

Statistical analysis of the received data proved that the average EIP values in control subjects differ significantly from the average EIP values in both people with plaque psoriasis (NEUT-RI, *p* < 0.0001, NEUT-GI, *p* < 0.0001, and RE-LYMPH, *p* < 0.0001) and with psoriatic arthritis (RE-LYMPH, *p* = 0.002) (see Figure 1). There were no significant differences in the values of these parameters between the group of patients with psoriasis vulgaris and the group with psoriatic arthritis. Furthermore, EIP (e.g., reactive lymphocyte count or percentage) combined with conventional inflammatory markers (e.g., WBC count) enabled accurate identification of individuals with psoriasis differentiating between them and healthy controls (see Figure 2).

The analysis of the dependence of changes in EIP on the severity of psoriasis was carried out in 29 patients who were classified using the PASI, BSA, and DLQI scales. A statistically significant correlation was found between the values of the RE-LYMPH (10^3^/*μ*l) parameter and the severity of the disease expressed in the PASI (*p*=0.045) and BSA (*p*=0.045) scales (see Table 3, line 3). In the case of the remaining parameters, no statistically significant relationships were found.

Moreover, a significant dependence of the size of the parameters NEUT-RI (*p*=0.021), NEUT-GI (*p*=0.011), and RE-LYMPH% (*p*=0.042) on the treatment used was found. The highest values of NEUT-RI, NEUT-GI, and RE-LYMPH% are observed with the use of local treatment and treatment with cyclosporine, acitretin, and methotrexate only. NEUT-RI values less than 50 units (FI, standard scope: 39.8–51.0 FI), NEUT-GI less than 150 units (SI, standard scope: 142.8–159.3 SI), and RE-LYMPH% less than 10% (standard scope: <5%) were observed during treatment with immunomodulatory drugs (ixekizumab, ustekinumab, secukinumab, golimumab, or adelimumab). There was no significant dependence of the values of parameters from the EIP group with the duration of treatment.

The analysis of interferon gamma and angiotensin II levels did not allow to show statistically significant relationships between the increase in concentrations of these parameters and the changes in haematological parameters of EIP (see Table 1, lines 15 and 16). However, concerning the risk assessment of coexisting diseases, e.g., from the cardiovascular system, there is a similar dynamic of changes in these parameters to changes in the NEUT-RI level and the absolute value of RE-LYMPH.

### 3.2. Discussion

The classical diseases coexisting with moderate and severe psoriasis include a number of diseases of inflammatory origin, such as psoriatic arthritis, inflammatory bowel disease, psychiatric and psychological problems, or uveitis [2]. In recent years, metabolic syndrome has also been included in the diseases accompanying psoriasis [5]. The exact underlying mechanism of metabolic syndrome in psoriasis has not been explained to date. Currently, many studies are devoted to the search for common pathogenetic mechanisms. High concentrations of certain cytokines, such as TNF alpha and IL-6, in individuals with psoriasis lead, i.a., to stimulation of the HPA axis and thus contribute to the development of central obesity, hypertension, and insulin resistance, while cytokines secreted in these conditions and plasminogen activation inhibitor, as well as some adipokines, maintain chronic inflammation and make skin lesions to become more severe and persist [5]. Therefore, it seems justified to analyse the development and severity of inflammation in individuals with psoriasis more thoroughly.

In the laboratory assessment of inflammation, it has recently been possible to use new haematological parameters informing about the activation status of immune system cells. These parameters are available in haematological analysers as Extended Inflammation Parameters (EIP) [8]. They illustrate the number of inflammatory cells in the body, i.e., activated lymphocytes—RE-LYMPH and AS-LYMPH—and neutrophil activation status—NEUT-RI and NEUT-GI [15]. The authors have decided to check the usefulness of the EIP group in the assessment of the activation of immune system cells in patients with diagnosed psoriasis. It turns out that using EIP could make it possible to easily and quickly assess the type of body's activated inflammatory response (cellular or humoral), as well as the severity of inflammation developing in psoriasis and the risk of coexisting diseases, such as cardiovascular diseases, which involve prolonged activation of immune system cells.

Several years ago, Zimmermann et al. [20] and Cornet et al. [9] suggested in their studies a statistically significant relationship between increased values of EIP and systemic inflammation (*p* < 0.05). New haematological markers of inflammation were also assessed by Henriot et al. [8]. Based on the review of these works and own preliminary studies [15, 21], the parameters of the EIP group were measured in individuals with psoriasis and healthy controls. Higher statistically significant results of EIP were observed in individuals with psoriasis than in healthy controls (*p* < 0.0003). Moreover, with regard to the RE-LYMPH (10^3^/*μ*l) parameter, this increase correlates with the severity of the disease expressed on the PASI and BSA scales. These results were confirmed by previous study reports [22–24]. A significant increase in the number of activated lymphocytes and neutrophils in psoriasis development results from the interaction of keratinocytes, i.a., IL-23 and IL-17. Interleukin 17, secreted by T17 lymphocytes, neutrophils, and NK cells, stimulates keratinocytes to produce proinflammatory cytokines and also drives and sustains the recruitment and activation of T lymphocytes and neutrophils that infiltrate the dermis [23]. The changes in the EIP group perfectly illustrate this mechanism. A higher EIP value was noted in the group of individuals with plaque psoriasis than in the group of individuals with psoriatic arthritis. This may be due to the fact that individuals with psoriatic arthritis are much more often subjected to immunosuppressive treatment, e.g., with methotrexate, which significantly reduces the severity of inflammation [25]. In this respect, the results of the dependence of changes in EIP on the treatment used seem interesting. The results (NEUT-RI: *p*=0.021, NEUT-GI: *p*=0.011, and RE-LYMPH%: *p*=0.042) suggest a reduction in the activation of neutrophils and lymphocytes in the course of treatment of psoriasis with immunomodulating drugs and may constitute the basis for further research.

Angiogenesis plays a fundamental role in psoriatic inflammation [26]. Interferon gamma and angiotensin II may be modulators of this process in psoriasis. In the study, we observed not only a slight increase in these parameters in individuals with diagnosed psoriasis compared to the control group but also a similar dynamic of changes in INF*γ* and ANGII levels compared to the EIP group. This confirms that activated cells of the immune system (neutrophils and T lymphocytes) stimulate the inflammatory response in psoriasis [24].

## 4. Conclusions

In conclusion, it should be noted that both the available literature and the studies covered by this article clearly indicate that the determination of complete blood count enriched with EIP in individuals with psoriasis may contribute to the improvement of the diagnostic interpretation of a patient's test results. This is of particular importance concerning the recognition of risk factors for coexisting diseases by dermatologists. Most often, psoriasis is diagnosed in young people, whose risk of diseases coexisting with psoriasis resulting from prolonged inflammation can be predicted. A dermatologist, knowing the patient's medical history, having history and laboratory test results, including complete blood count results with the assessment of EIP, will be able to diagnose comorbidities more easily, assess the risk of their development, and thus minimize their negative effects. To our knowledge, this is the first study to assess EIP in psoriasis. However, prospective studies of these parameters in a larger group of patients with psoriasis are needed.

## Figures and Tables

**Table 1:** Characteristics of the study group and the control group.

		Total *N* = 61, range (*N*) (average (%))	
	*Demographic data*		
		Study group = 31	Control group = 30
1.	Age (years)	19–71 (43)	21–72 (49)
2.	Male gender	22 (70.9)	12 (40,0)

	*Clinical data*		
3.	Smoking	3 (9.7)	5 (16.7)
4.	Time since psoriasis diagnosis (years)	1.5–39.0 (15.0)	—
5.	Accompanying chronic diseases*∗*	18 (58.1)	10 (33.3)
6.	Cancers	0	0
7.	Immunosuppressive treatment	22 (70.9)	0

	*Laboratory data*		
8.	CRP (mg/l)	0.6–17.0 (4.39)	1.0–8.9 (3.06)
9.	ESR (mm/h)	23–98 (36.5)	2–19 (1.23)
10.	WBC (10^3^/*μ*l)	4.46–10.59 (7.13)	3.44–9.78 (6.26)
11.	NEUT (10^3^/*μ*l)	1.99–8.40 (4.09)	1.56–5.78 (3.54)
12.	NEUT (%)	38.1–79.4 (56.3)	32.5–61.0 (52.1)
13.	LYMPH (10^3^/*μ*l)	1.07–3.52 (2.20)	1.15–2.94 (2.01)
14.	LYMPH (%)	14.3–49.5 (31.6)	18.1–41.1 (30.8)
15.	INF*γ* (pg/ml)	0.64–5.70 (1.95)	0.15–2.51 (1.84)
16.	ANG II (pg/ml)	25.44–125.70 (54.4)	25.98–68.52 (46.15)

^*∗*^Including pulmonary sarcoidosis, nephrolithiasis, inflammation of the duodenal mucosa, bronchial asthma, hypertension, hypercholesterolaemia, urinary tract infection, gout, diabetes, chronic heart failure, obesity, depression, hypothyroidism, degenerative spine disease, arthrosis, and juvenile acne. *N*: the number of all variables; CRP: C-reactive protein; ESR: erythrocyte sedimentation rate; WBC: white blood cells; NEUT: neutrophils; LYMPH: lymphocytes; INF*γ*: interferon gamma; ANGII: angiotensin II.

**Table 2:** Descriptive characteristics of parameters belonging to the EIP group in the study groups and the control group.

	Tested parameter	Group	*N*	Average	Median	Min	Max	SD
1.	NEUT-RI (FI)	Control group	30	44.01	43.50	40.30	50.10	2.10
		A group of patients with plaque psoriasis	25	51.28	51.70	41.80	59.70	5.45
		A group of patients with psoriatic arthritis	6	46.80	45.90	39.20	54.80	5.96

2.	NEUT-GI (SI)	Control group	30	146.68	146.20	137.80	157.00	4.25
		A group of patients with plaque psoriasis	25	151.44	152.10	144.20	158.40	3.52
		A group of patients with psoriatic arthritis	6	150.42	149.85	142.10	159.70	6.21

3.	RE-LYMPH (10^3^/*μ*l)	Control group	30	0.04	0.03	0.01	0.08	0.02
		A group of patients with plaque psoriasis	25	0.29	0.28	0.05	0.98	0.22
		A group of patients with psoriatic arthritis	6	0.15	0.15	0.04	0.34	0.12

4.	AS-LYMPH (10^3^/*μ*l)	Control group	30	0.00	0.00	0.00	0.00	0.00
		A group of patients with plaque psoriasis	25	0.00	0.00	0.00	0.00	0.00
		A group of patients with psoriatic arthritis	6	0.001	0.00	0.00	0.04	0.02

*N*: the number of all variables; SD: standard deviation; NEUT-GI: neutrophil granularity intensity; NEUT-RI: neutrophil reactive intensity; RE-LYMPH: reactive lymphocytes; AS-LYMPH: antibody-secreting reactive lymphocytes.

**Table 3:** Relationship between the level of haematological parameters of EIP and the stage of the disease.

	Tested parameter	Group	*N*	PASI	BSA	DLQI scale
1.	NEUT-RI (FI)	A group of patients with psoriasis	29	*p*=0.289	*p*=0.417	*p*=0.988
2.	NEUT-GI (SI)	A group of patients with psoriasis	29	*p*=0.789	*p*=0.872	*p*=0.401
3.	RE-LYMPH (10^3^/*μ*l)	A group of patients with psoriasis	29	*p*=0.045	*p*=0.045	*p*=0.325

*N*: the number of all variables; *p*: statistical significance; PASI: psoriasis area and severity index; BSA: body surface area; DLQI scale: dermatology life quality index; NEUT-GI: neutrophil granularity intensity; NEUT-RI: neutrophil reactive intensity; RE-LYMPH: reactive lymphocytes; AS-LYMPH: antibody-secreting reactive lymphocytes.

## Data Availability

The data used in this study are sensitive patient data, partly belonging to Independent Public Clinical Hospital No. 1 in Lublin. For this reason, they can only be shared with the consent of the hospital. In this case, please contact the hospital's data protection officer or the director of the hospital for medical affairs (ul. Staszica 16, 20-081 Lublin, szpital@spsk1.lublin.pl).
